# Asthma medication adherence, control, and psychological symptoms: a cross-sectional study

**DOI:** 10.1186/s12890-024-02995-x

**Published:** 2024-04-19

**Authors:** Abdullah A. Alqarni, Abdulelah M. Aldhahir, Rayan A. Siraj, Jaber S. Alqahtani, Dana A. Alghamdi, Sarah K. Alghamdi, Abeer A. Alamoudi, Majduleen A. Mohtaseb, Mansour Majrshi, Abdulkareem A. AlGarni, Omaima I. Badr, Hassan Alwafi

**Affiliations:** 1 Department of Respiratory Therapy, Faculty of Medical Rehabilitation Sciences, King Abdulaziz University, Jeddah, Saudi Arabia; 2 Respiratory Therapy Unit, King Abdulaziz University Hospital, Jeddah, Saudi Arabia; 3 Respiratory Therapy Department, Faculty of Applied Medical Sciences, Jazan University, Jazan, Saudi Arabia; 4 Department of Respiratory Care, College of Applied Medical Sciences, King Faisal University, Al-Ahsa, Saudi Arabia; 5 Department of Respiratory Care, Prince Sultan Military College of Health Sciences, Dammam, Saudi Arabia; 6 National Heart and Lung Institute, Imperial College London, London, UK; 7 Respiratory Medicine, Royal Brompton Hospital, London, UK; 8 King Abdulaziz Hospital, The Ministry of National Guard Health Affairs, Al Ahsa, Saudi Arabia; 9 King Saud bin Abdulaziz University for Health Sciences, College of Applied Medical Sciences, Al Ahsa, Saudi Arabia; 10 Department of Chest Medicine, Faculty of Medicine, Mansoura University, Mansoura, Egypt; 11 Department of Pulmonary Medicine, Al Noor Specialist Hospital, Mecca, Saudi Arabia; 12 Department of Clinical Pharmacology and Toxicology, Faculty of Medicine, Umm Al-Qura University, Makkah, Saudi Arabia

**Keywords:** Asthma, Depression, Anxiety, Adherence to inhaler, Asthma control, Adherence to controller therapies

## Abstract

**Background:**

Nonadherence to therapies and psychological disorders are associated with poor asthma control. This study aims to assess the prevalence of anxiety and depressive symptoms, asthma control, and adherence to inhalers and to investigate whether there is an association of anxiety and depressive symptoms with adherence to inhalers and asthma control.

**Methods:**

We measured anxiety and depressive symptoms using the Hospital Anxiety and Depression Scale in patients with asthma. Asthma Control Test and the 10-Item Test of Adherence to Inhalers Scale were used to assess levels of asthma control adherence to inhalers, respectively. Univariate and multivariate regression models assessed the associations of anxiety and depressive symptoms with adherence to inhalers and asthma control.

**Results:**

A total of 287 patients completed the study, of whom 72% were female. The mean ± SD age and body mass index of our study population were 44 ± 13 years and 29 ± 7.2 kg/m^2^, respectively. Poor adherence to inhaler use was highly prevalent (49.8%; 95% CI: 43.8 to 55.7). The prevalence of anxiety, depression and poor asthma control was 27.2% (95% CI: 22.1 to 32.7), 20.9% (95% CI: 16.3 to 26.1), and 22.7% (95% CI: 17.9 to 27.9), respectively. We found a negative relationship between asthma control and anxiety, and depressive symptoms (adjusted β: -0.25; 95% CI: -0.36 to -0.14; *p* < 0.001 and adjusted β: -0.29; 95% CI: -0.40 to -0.18; *p* < 0.001, respectively). A negative relationship was also observed between adherence to inhalers and anxiety and depressive symptoms (adjusted β: -0.34; 95% CI: -0.46 to -0.22; *p* < 0.001 and adjusted β: -0.36; 95% CI: − 0.48 to − 0.24; *p* < 0.001, respectively).

**Conclusions:**

The high prevalence of uncontrolled asthma symptoms and poor adherence to inhalers and their impact on anxiety and depression levels among patients with asthma point to the need for early screening for psychological symptoms and recognition of nonadherence as part of asthma assessment and management plan in primary care in Saudi Arabia to avoid further worsening of asthma symptoms. Further studies are needed to explore the effectiveness of specific psychoeducational interventions and investigate the long-term impact of early psychological symptom detection on asthma outcomes.

**Supplementary Information:**

The online version contains supplementary material available at 10.1186/s12890-024-02995-x.

## Introduction

Asthma, an airway inflammation disease, is one of the most debilitating chronic diseases impacting people worldwide and is a common disorder with a variety of etiologies, including environmental and genetic variables. In 2019, the World Health Organization estimates that asthma affected 262 million people and caused 461.000 deaths globally [1]. In Asia, cases of asthma have been increasing over time, with a prevalence ranging from 1 to 10.3% [2]. Although the prevalence of asthma in Saudi Arabia is under-investigated, it has been reported that overall prevalence rates of asthma symptoms in Saudi adults ranges from 14.2 to 18.2% [3, 4]. It should be noted that symptoms of asthma might vary from person to person. A common symptom of asthma includes, shortness of breath, chronic cough, particularly at night. The worsening of asthma symptoms may lead to several consequences, including poor quality of life, frequent visits to the emergency room, and hospital stays [5–8].

Although asthma is not a curable disease, the use of pharmacologic therapies (e.g., inhaled corticosteroid (ICS) with or without long-acting beta-agonist (LABA)) is considered the mainstay in the prevention of the frequent worsening of asthma symptoms and hospital admission of patients with unstable asthma [5]. Good adherence to these controller therapies is associated with better clinical outcomes in asthma patients, including better disease management and reduction in severe asthma exacerbations [9]. Globally, the reported rates of nonadherence to asthma medication are high [10]. For instance, levels of nonadherence in Kuwait, Northwestern Ethiopia, Bangladesh, Tanzania, and Northern Ireland were reported to be 82.6% [11], 86.1% [12], 86% [13], 60.3% [14], and 88% [15] of patients with asthma, respectively. In Saudi Arabia, the exact prevalence of nonadherence has not been well assessed in patients with asthma, likely due to health system issues such as time constraints and heavy workloads which prevent pulmonologists and respiratory therapists from assessing level of adherence to inhalers use during routine outpatient visits. Nonetheless, it has been shown that almost 50% of patients with chronic obstructive pulmonary disease (COPD) are poorly adherent to the use of their medications [16]. Different factors may contribute to patient nonadherence to medication across a range of chronic diseases, one of which is the presence of psychological disorders [17].

A recent national screening for anxiety and depression was conducted among the general Saudi population and showed that the prevalence of people at risk of depression and anxiety was 12.7% and 12.4%, respectively [18]. Symptoms of anxiety and depression have also been reported in patients with asthma [19], particularly in those who are corticosteroid dependent [20]. Although the prevalence of anxiety and depression among patients with asthma in Saudi Arabia has not been well reported, these symptoms vary globally, ranging from 27 to 36% [21–23] and 11–14% [23–25], respectively. More importantly, it has been suggested that uncontrolled asthma and poor adherence to a maintenance medication regimen dramatically enhance psychological morbidity among patients with asthma and subsequently lead to increased exacerbation rates [26].

Given that early recognition and interventions are important to effectively address nonadherence to controller medication and psychological disorders, it is essential to study the extent to which psychological distress impacts medication adherence and asthma control among adults with asthma. Therefore, this study aimed to 1) assess the prevalence of adherence to inhalers, asthma control, and anxiety and depression levels among patients with asthma, 2) investigate whether there is an association of anxiety and depressive symptoms with adherence to inhalers and asthma control; and 3) evaluate whether the nonadherence to medication and poor asthma control can affect levels of anxiety and depressive symptoms.

## Methods

### Study design and setting

This cross-sectional study was carried out between September 2022 and June 2023 at King Abdulaziz University Hospital and a Ministry of Health hospital located in the western region of Saudi Arabia.

### Study population

Patients who agreed to participate and signed the informed consent form were included in this study. Patients were eligible for participation only if they had a multidisciplinary team-confirmed diagnosis of asthma based on current nationally accepted criteria [27]. Diagnostic criteria for asthma include the presence of variable respiratory symptoms history together with a confirmed variable expiratory airflow limitation (defined as increase in forced expiratory volume in one second (FEV_1_) of > 12% and > 200 mL). In addition, patients were included in the study if they were on asthma maintenance therapy (ICS with or without LABA), had never smoked and were aged 18 or older. Exclusion criteria included patients with a smoking history, who were not on maintenance asthma inhalers, who were aged < 18 years, and who could not complete the study.

### Spirometry parameters

Recent spirometry parameters, including forced vital capacity (FVC), FEV_1_, ratio of FEV_1_/FVC, forced expiratory flow at 25% and 75% of the pulmonary volume (FEF 25–75%) and peak expiratory flow (PEF)) were collected from the medical records of asthmatic patients who visited the pulmonary clinic within the last 6 months before this study was carried out. The spirometry tests were conducted as part of routine care in the pulmonary clinic by a well-trained pulmonary function technician and reviewed by a pulmonologist and a consultant respiratory therapist in accordance with the current American Thoracic Society/European Respiratory Society guidelines [28].

### Data collection procedures and tools

This study was conducted with outpatients with confirmed asthma diagnoses who had scheduled visits and consultations with specialists at two pulmonary clinics. Data were collected by three senior respiratory therapists with four respiratory therapy interns under the supervision of two consultant respiratory therapists and a pulmonologist. The study aims were explained to the patients, after which data collectors invited the patients to complete three patient-reported outcome measures, namely, the Asthma Control Test (ACT), the Hospital Anxiety and Depression Scale (HADS), and the Test of Adherence to Inhalers (TAI). The validity and reliability of ACT and HADS questionnaires have been previously established within the Saudi Arabian population [29, 30]. Before the start of this study, content and face validity were assessed for TAI questionnaire. Additionally, TAI questionnaire was piloted to 10 patients not included in the study and the overall Cronbach’s alpha reliability coefficient was 0.71, demonstrating a reliable tool. The relevant patient characteristics, demographic data and spirometry results of the patients included in this study were collected from patient medical records.

### Patient-reported outcome measures

#### Asthma control

The ACT comprises 5 questions that evaluate a 4-week recall of symptoms and daily functioning. The score for each question is scaled from 1 (totally uncontrolled) to 5 (fully controlled), where 1 means the patient experiences asthma symptoms all the time and 5 means the patient does not experience symptoms at all. The scores on each question are summed and range from 5 to 25. Higher scores [20–25] indicate better asthma control. Scores of 15–19 are considered “not well controlled,” and 5–14 indicate “very poorly controlled” [31].

#### Anxiety and depression

The 14-item HADS constitutes 14 questions divided equally for depression and anxiety. Since each item is evaluated between 0 (no impairment) and 3 (severe impairment) on a 4-point severity scale, the possible scores range from 0 to 21 for anxiety and 0 to 21 for depression. Scores of 7 or less on either scale suggest a normal degree of anxiety or depression, while scores of 8–10 indicate borderline. A score of 11–21 is considered abnormal anxiety or depression [32].

#### Adherence to inhalers

The TAI is used specifically to evaluate the level of inhaler adherence in patients with respiratory diseases, including asthma. The TAI is a validated tool that provides an accurate indicator of patient adherence to the inhaler and is a patient-focused questionnaire measuring how well asthma or COPD patients adhere to their inhaler regimens [33]. The TAI has 10 items, with the score of each item ranging from 1 (worst compliance) to 5 (best compliance). The TAI provides a total sum score between 10 (minimum) and 50 (maximum), which can determine the degree of patient adherence to asthma inhalers: good (50 points), intermediate (46–49 points), or poor (≤ 45 points). Before the start of the study, written permission was obtained from the copyright owners of the TAI questionnaire [33].

### Ethical consideration

Before the start of this study, we obtained ethical approval from an independent research committee at King Abdulaziz University, Saudi Arabia (No 407 − 22).

### Statistical analysis

The data were analyzed using GraphPad Prism (Version 9). The normality of the data was graphically assessed using histogram to determine the appropriate statistical tests. Categorical variables are presented as percentages and frequencies, while arithmetic means ± SDs were used for normally distributed continuous variables. Parametric tests such as one-way ANOVA and independent t-test were performed to determine whether there were differences in scores between the control (e.g., well-controlled asthma or good adherence to therapies) and specific groups of asthma control or medication adherence. The correlation of adherence to inhaler use and asthma control with both anxiety and depression was determined using Pearson’s correlation coefficient. Univariate and multivariate regression models were also performed to determine the factors associated with depressive and anxiety symptoms, with adjustment for potential confounders (age, gender, and body mass index (BMI)). P *<* 0.05 was regarded as statistically significant.

## Results

### Patient characteristics

**Fig. 1:**
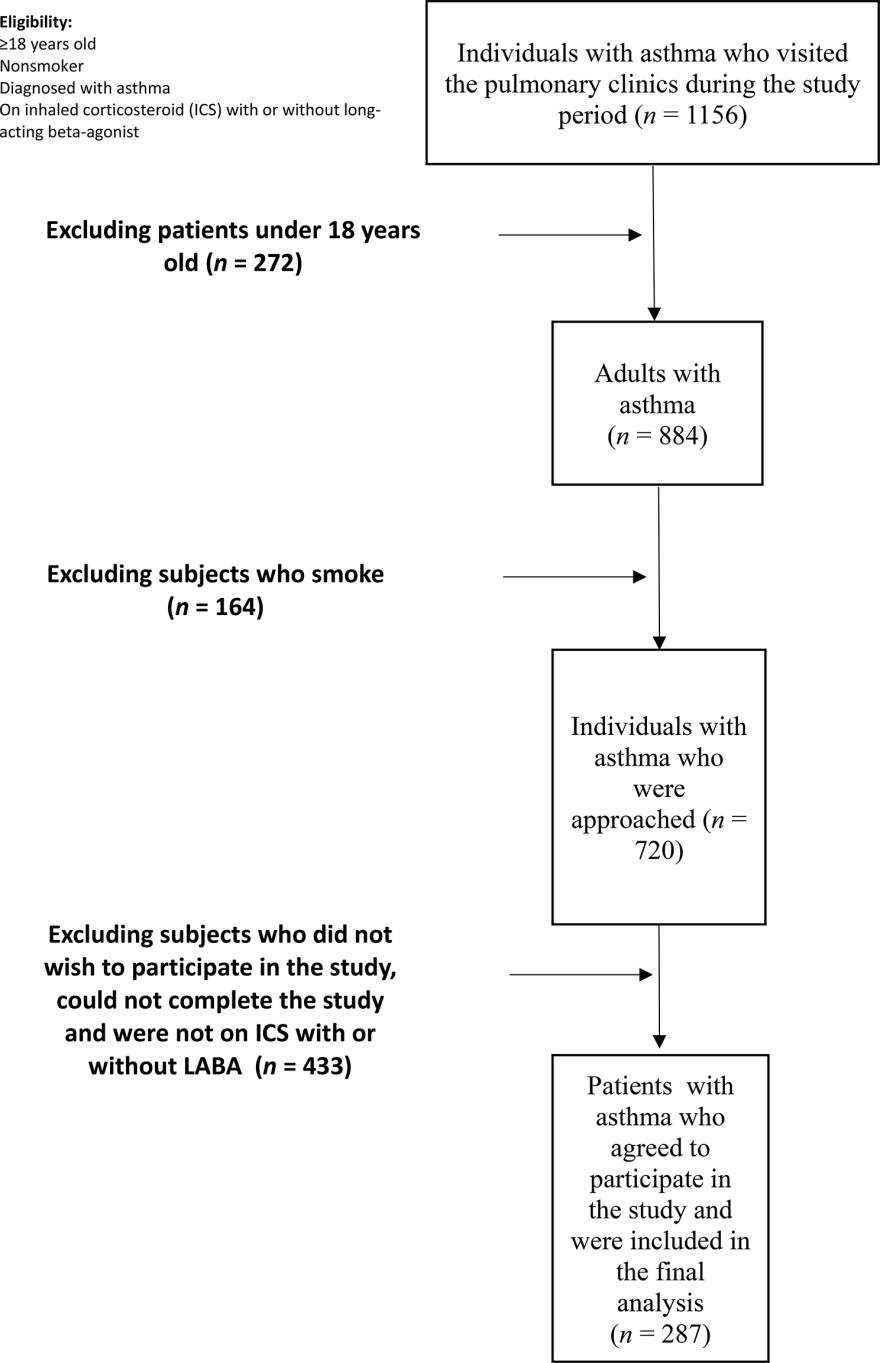
Flow chart of the study

We identified 1,156 patients with asthma who visited the studied pulmonary clinics for routine follow-up during the data collection process. After we excluded those aged < 18 years old, those who did not have a history of smoking, and those who were not on asthma controller therapies, 720 adult individuals with asthma were approached. Of these, only 287 patients met our inclusion criteria and were included in the final analysis (Fig. 1).

The mean ± SD age and BMI of our study population were 44 ± 13 years and 29 ± 7.2 kg/m^2^, respectively. The mean ± SD scores of anxiety, depression, asthma control and adherence to inhaler were 4.8 ± 5, 4.5 ± 5, 18.0 ± 5, and 44 ± 5.3, respectively. Some other demographic and clinical characteristics of the study participants are described in Table 1. Further details can be found in the supplementary file.

**Table 1:** Patient characteristics (*n* = 287)

Variable			Total (*n* = 287)
Age (years)			44 ± 13
Female, *n* (%)			207 (72%)
BMI (kg/m^2^)			29 ± 7.2
Depression score (assessed using the HADS tool)			4.5 ± 5
Anxiety score (assessed using the HADS tool)			4.8 ± 5
Adherence to Inhaler score (assessed using the TAI tool)			44 ± 5.3
Asthma control score (assessed using the ACT tool)			18 ± 5
Spirometry parameters			
FEV_1_ (% predicted) FVC (% predicted) PEF (% predicted) FEF _25−75%_ (% predicted) FEV_1_/FVC ratio (% predicted)			68 ± 20 80 ± 16 77 ± 23 66 ± 29 76 ± 16

Data are represented as the mean ± SD unless otherwise stated. BMI: body mass index: ACT: Asthma Control Test. HADS: Hospital Anxiety and Depression Scale. TAI: Test of Adherence to Inhalers. FVC: forced vital capacity. FEV_1_: forced expiratory volume in one second. FEF _25–75%_: forced expiratory flow at 25% and 75% of the pulmonary volume. PEF: peak expiratory flow

### The prevalence of asthma control, symptoms of anxiety and depression, and adherence to inhaler use

We found that 78 (27.2%, 95% CI: 22.1 to 32.7) of our study population had symptoms of anxiety, while 60 (20.9%, 95% CI: 16.3 to 26.1) reported depressive symptoms (Table 2). Then, the 5-point questionnaire of the ACT tool was used to assess asthma control among our study participants. Asthma symptoms were uncontrolled in 65 (22.7%, 95% CI: 17.9 to 27.9) subjects (Table 2). Next, we investigated whether the patients were adherent to inhaler use and found that half of our study population (49.8%, 95% CI: 43.8 to 55.7) was poorly adherent to their inhaler use (Table 2).

**Table 2:** The level of anxiety, depression, asthma control and adherence to inhaled asthma medication among patients with asthma (*n* = 287)

Level of anxiety	Frequency (%)	95% CI
Abnormal or Borderline (defined as a HADS score of 8 to 21)	78 (27.2%)	22.1 to 32.7
Level of depression	Frequency (%)	
Abnormal or Borderline (defined as a HADS score of 8 to 21)	60 (20.9%)	16.3 to 26.1
Level of asthma control	Frequency (%)	
Uncontrolled (defined as an ACT score of 14 to 5)	65 (22.7%)	17.9 to 27.9
Level of adherence to inhaler	Frequency (%)	
Poor adherence (defined as a TAI score of ≤ 45)	143 (49.8%)	43.8 to 55.7

*Abbreviations* ACT: asthma control test, HADS: hospital anxiety and depression scale, TAI: Test of Adherence to Inhalers. CI: confidence interval

### The association of symptoms of anxiety and depression with asthma control and adherence to inhaled therapy use

**Fig. 2:**
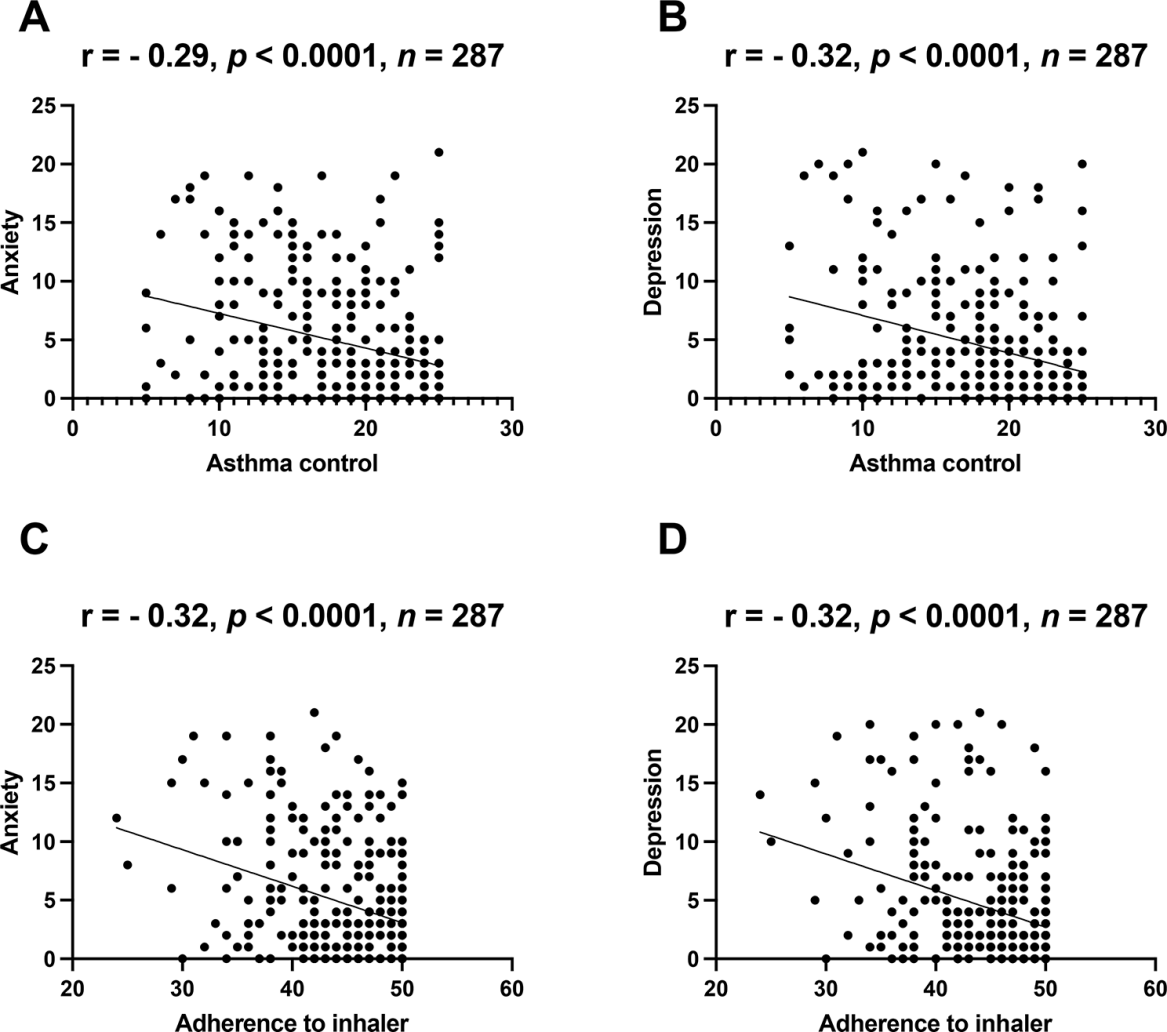
The association of symptoms of anxiety and depression with asthma control and adherence to inhaler use among patients with asthma. The asthma control test (ACT) was used to assess the levels of asthma control. The levels of adherence to inhaler use were assessed using the Test of Adherence to Inhalers (TAI) tool. Anxiety and depression were assessed using the hospital anxiety and depression scale (HADS)

Pearson correlation analyses were performed to determine whether symptoms of anxiety and depression are associated with asthma control and adherence to inhaled asthma medications. Our results showed that anxiety was negatively associated with asthma control (r = −0.29, p < 0.0001) (Fig. 2A) and adherence to inhaler use (r = −0.32, p < 0.0001) (Fig. 2C). Similarly, we found negative associations of depression with asthma control (r = −0.32, p < 0.0001) and adherence to inhaler use (r = −0.32, p < 0.0001) (Fig. 2B-D).

Next, univariate and multivariate regression models were performed to assess the associations of adherence to inhaler use and asthma control with both anxiety and depression (Table 3). A unit change (a 1-point increase) in adherence to inhaler use was associated with a decrease in both anxiety and depression symptoms (β: -0.33; 95% CI: -0.45 to -0.22; p < 0.001 and β: -0.35; 95% CI: -0.47 to -0.23; p <0.001, respectively) (Table 3). These associations remained the same even after adjusting for age, sex, and BMI. As shown in Table 3, negative associations of asthma control were also observed with both anxiety and depressive symptoms, even after adjusting for confounders. These findings indicate that the more likely patients with asthma adhere to the use of their inhalers or have well controlled asthma symptoms, the less likely they experience anxiety and depressive symptoms.

**Table 3:** An analysis of the associations of anxiety and depression with adherence to inhaler use and asthma control

Dependent variable: Anxiety							
	**β (95% CI)**		*Adjusted β (95% CI)			***P*** **value**	
Adherence to inhaler use– per 1 score	-0.33 (-0.45 to -0.22)		-0.34 (-0.46 to -0.22)			<0.001	
Asthma control– per 1 score	-0.28 (-0.39 to -0.17)		-0.25 (-0.36 to -0.14)			<0.001	
**Dependent variable: Depression**							
	**β (95% CI)**		*Adjusted β (95% CI)			***P*** **value**	
Adherence to inhaler use– per 1 score	-0.35 (-0.47 to -0.23)		-0.36 (-0.48 to -0.24)			<0.001	
Asthma control– per 1 score	-0.32 (-0.43 to -0.21)		-0.29 (-0.40 to -0.18)			<0.001	

*Adjusted for age, sex, and body mass index

### The impact of poor asthma control and nonadherence to inhaled controller medications on anxiety and depressive symptoms

**Fig. 3:**
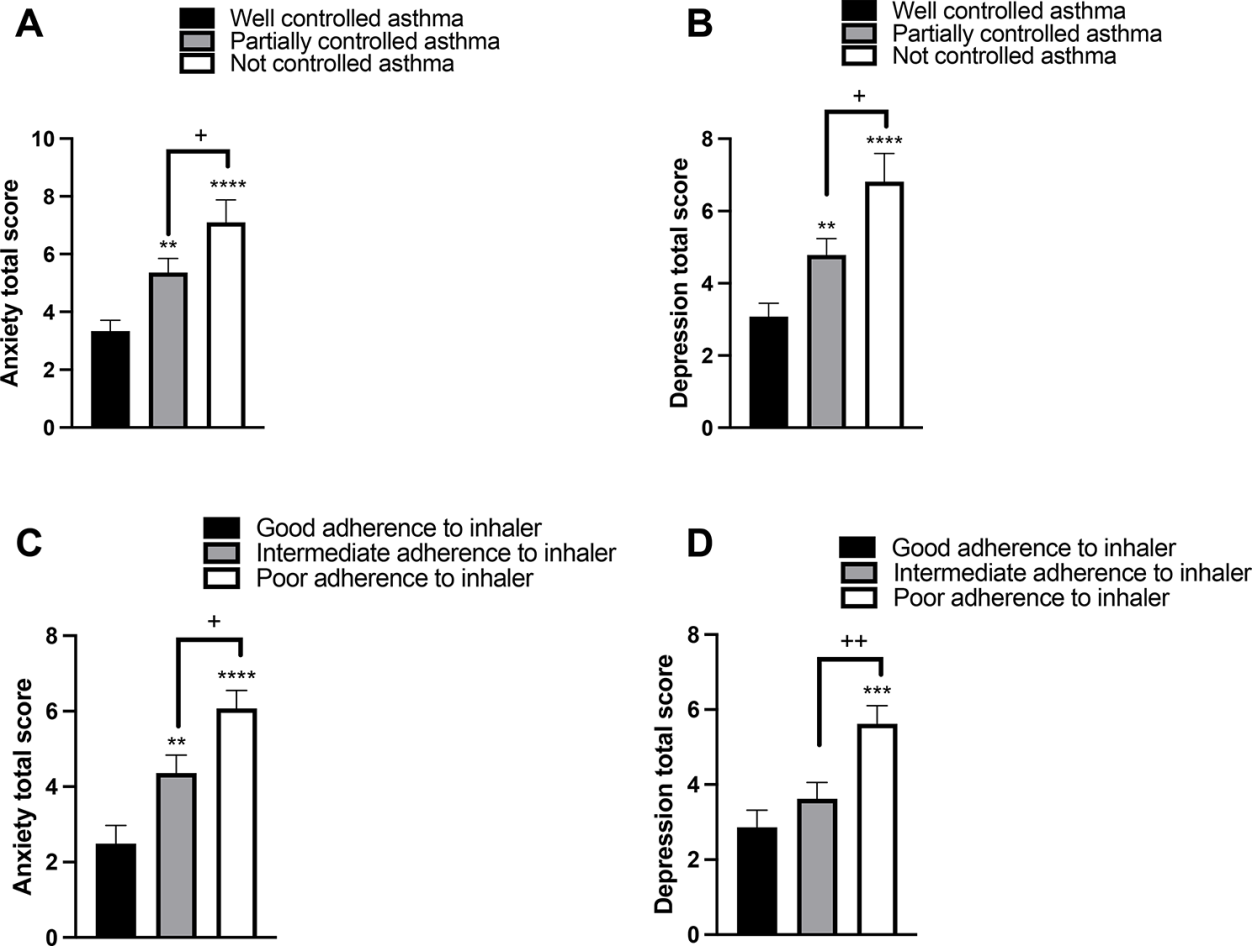
The impact of poor asthma control and nonadherence to asthma controller use on anxiety and depression levels among patients with asthma. Patients were divided into three groups based on the levels of asthma control: uncontrolled (an asthma control test (ACT) score of 14 to 5, *n* = 131), partially controlled (an ACT score of 19 to 15, *n* = 91), and fully controlled (an ACT score of 20 to 25, *n* = 65). Then, the levels of anxiety and depression were assessed using the hospital anxiety and depression scale (HADS) (**A** and **B**). Patients were also classified into three groups based on their level of adherence to inhaler use: good (a Test of Adherence to Inhalers (TAI) score of 50, *n* = 59), intermediate (a TAI score of 46 to 49, *n* = 85), and poor (a TAI score of ≤ 45, *n* = 143) before levels of anxiety and depression were determined (**C** and **D**). The bars represent the standard errors of the mean. *P < 0.05, **P < 0.01, ***P < 0.001, ****P < 0.0001 compared with the control; +*P* < 0.05, ++*P* < 0.01 compared with partially controlled asthma/intermediate adherence to medication

We next assessed the extent to which poor asthma control and nonadherence to the use of asthma controller medications impact levels of anxiety and depression among the participants. The means of anxiety and depression in patients with well-controlled asthma were 3.3 ± 0.38 and 3.1 ± 0.37 HADS scores, respectively (Fig. 3A and B). We found that the levels of both anxiety and depression were significantly higher in asthmatic patients with uncontrolled asthma (5.4 ± 0.49 and 4.8 ± 0.45 HADS score, respectively) compared with patients with well-controlled asthma (*p* < 0.0001 for both) (Fig. 3A and B).

We then found that the mean anxiety and depression among patients with good adherence to inhaler use were 2.5 ± 0.48 and 2.9 ± 0.45 HADS scores, respectively (Fig. 3C and D). In patients with poor adherence to inhaler use, the anxiety and depression levels were found to be greater (6.1 ± 0.48 and 5.6 ± 0.48 HADS score) compared to the good adherence to inhaler use group (Fig. 3C and D). These findings suggest that asthmatic patients with poor asthma control and low adherence to inhalers are more likely to report increased levels of anxiety and depressive symptoms.

**Fig. 4:**
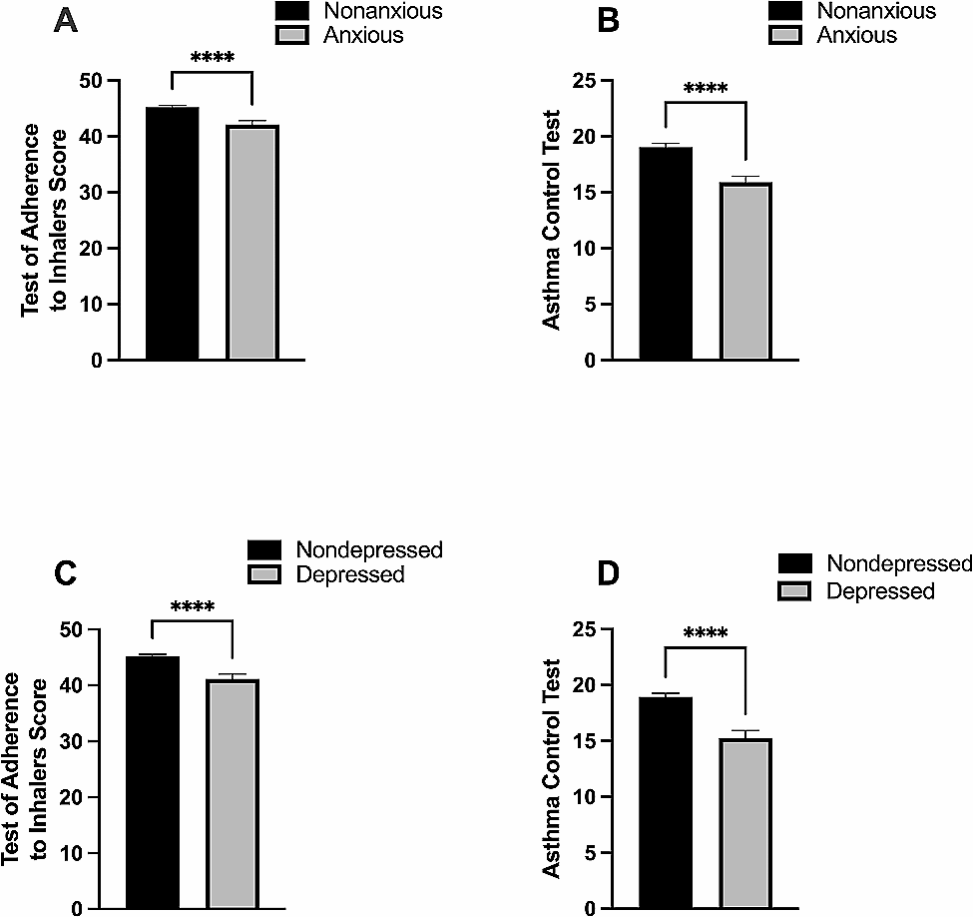
The impact of anxiety and depression on asthma control and adherence to asthma controller among patients with asthma. Patients were classified into two groups based on the levels of anxiety and depression (as assessed by the Hospital Anxiety and Depression Scale (HADS). Pateints with HADS score of 8 to 21 were considered anxious (*n* = 78) or depressed (*n* = 60), and those with HADS score of 0 to 7 were considered nonanxious (*n* = 209) or nondepressed (*n* = 227). Then, level of adherence to inhalers (**A** and **C**) and asthma control (**B** and **D**) were assessed using Test of Adherence to Inhalers (TAI) and Asthma Control Test (ACT), respectively. The bars represent the standard errors of the mean. ****P < 0.0001 compared with the nonanxious or nondepressed groups

Next, subjects were classified into two groups based on the levels of anxiety and depression and then level of adherence to inhalers and asthma control were assessed. The means of adherence to inhalers and asthma control levels in nonanxious group were 45 ± 0.33 and 19 ± 0.34, respectively and were significantly reduced in anxious group with asthma (42 ± 0.68 and 16 ± 0.55, respectively, *P* < 0.0001 for both) (Fig. 4A and B). Similarly, adherence to inhalers and asthma control levels were significantly lower in depressed patients with asthma 41 ± 0.84 and 15 ± 0.66, respectively compared with nondepressed group (45 ± 0.31 and 19 ± 0.32, respectively, *P* < 0.0001 for both) (Fig. 4C and D).

## Discussion

Our main findings demonstrated that poor adherence to inhaler use was highly prevalent (49.8%) among patients with asthma. In addition, we showed that there are negative associations of symptoms of anxiety and depression with poor asthma control and nonadherence to medications. More importantly, it was demonstrated that levels of anxiety and depression were increased in a dose-dependent manner in patients with uncontrolled asthma as well as those with poor adherence to therapies compared with patients with well-controlled asthma and good adherence to therapies. These findings suggest that asthmatic patients with anxiety and depression are more likely to report poor adherence to inhaler use. Some of the findings reported in the current study have been presented in the form of abstracts [34].

 Anxiety and depression are among the psychological comorbidities frequently seen in asthma [19]. We have previously reported that a low rate of screening asthmatic patients for depression in clinical practice in Saudi Arabia exists, mainly due to physicians’ limited knowledge and poor training in identifying and managing psychological comorbidities in asthma [35]. In the current study, we found that anxiety and depression symptoms were prevalent (27.2% and 20.9%, respectively). Although this prevalence has not been well assessed previously in Saudi Arabia, our findings are similar to previous studies reporting that the anxiety rate ranges globally from 27–36% [21–23], but this range slightly differs in studies among patients with asthma, with the depression rate being measured at between 11% and 14% [22–25]. The differences in the prevalence of depression between the findings observed in this study and those reported by others can be explained by the fact that some of these previous studies included adult and adolescent participants, and they only limited their study samples to those with persistent asthma [22]. Despite these slight differences in depression prevalence among patients with asthma, symptoms of anxiety and depression are still common. Thus, routine assessment for psychological disorders is needed to prevent worsening in patients with asthma symptoms, given that psychological disorders are underdiagnosed in patients with chronic diseases [36, 37] and that physicians in Saudi Arabia who are in direct contact with asthmatic patients do not usually screen for psychological disorders [35] in the clinical setting.

 Poorly controlled asthma is associated with an increased risk of exacerbation and frequent emergency visits [38]. Patients with uncontrolled asthma are at high risk of developing psychological disorders, such as depression and anxiety [39]. In the current study, we found that 22.7% of asthmatic patients had poorly controlled asthma. Although the majority of our study population had well or partially controlled asthma, we found a negative association between asthma control and anxiety and depression among them. More importantly, the levels of both depression and anxiety were found to be higher in patients with poorly controlled asthma than in those with well-controlled asthma. These findings are supported by previous studies showing that anxiety and/or depressive symptoms in asthma patients are associated with poor asthma control [40, 41] and impaired health-related quality of life [41]. Our findings, together with these observations, highlight the need for appropriate assessments for these comorbid conditions, which may help to reduce anxiety and enhance quality of life.

There is evidence to show that psychological disorders in asthma patients are also linked to serious clinical outcomes [40], higher use of rescue inhalers [42], and poor adherence to medications [43]. Our finding of poor adherence to inhaler use (49.8%) among the participants in the current study is consistent with previous studies demonstrating that from 30–70% of patients with asthma adhere poorly to asthma medications [10]. Although the exact reasons for patient nonadherence to medication, especially in patients with asthma, are unclear, the use of multiple inhaler devices has been previously reported to play a role in inducing low levels of adherence to medication in patients with COPD [44]. Several factors, such as forgetfulness, lack of knowledge or conscious decision-making, may contribute to poor levels of adherence to medication in patients with chronic lung diseases [45].

It should be noted that our findings demonstrated a negative association between symptoms of anxiety and depression and levels of adherence to medication in adults with asthma. This is supported by previous studies reporting that the presence of anxiety and depression can influence adherence to medication in children and elderly individuals with asthma [43, 46]. Furthermore, this is further strengthened by the fact that levels of anxiety and depression in the current study were increased in people with poor adherence to inhaled therapies compared with good adherence levels, suggesting that symptoms of anxiety and depression are likely to increase when asthma is not well controlled, as well as in asthmatic patients who are poorly adherent to the use of their inhalers. Taken together with these observations, our findings suggest that poor control of asthma symptoms and nonadherence to the use of controller therapies are associated with the presence of psychological disorders in patients with asthma. Thus, early recognition of nonadherence to therapies, as well as identifying the exact type of noncompliance by implementing optimal patient education programs, are needed to apply the right corrective measures to improve levels of adherence, thereby leading to improvement in asthma symptoms and overall quality of life.

### Strengths and limitations

 This study has several strengths. This is the first study to determine the levels of adherence among Saudi patients with asthma to medications, particularly concerning asthma maintenance inhaler use, and assess the impact of nonadherence levels to inhaled therapies on anxiety and depression. In the present study, we only included patients with a multidisciplinary team-confirmed diagnosis of asthma based on current internationally and nationally accepted criteria. Smokers with asthma were excluded from this study due to the difficulty in separating asthma from COPD in smokers. It should be noted that this study is not without limitations. First, the cross-sectional nature of this study limits the possibility of assessing any causality. Hence, caution should be taken in interpreting our study findings as correlation does not imply causation. Second, it has been reported that socioeconomic factors are associated with mental health disorders [47] and poor medication adherence [48], it was difficult in the current study to assess the socioeconomic status of the study participants, as in Saudi society, generally, patients prefer not to declare their family income or levels of education. Third, patient self-reporting tools were used to assess the levels of adherence to inhaled therapies, which may overestimate adherence to therapy use [49]. The use of subjective methods together with objective assessment methods (e.g., electronic monitoring systems and measurement of drug/metabolite levels) should be considered in future studies to better assess level of adherence to inhalers among patients with asthma.

 Our study has practical implications. Healthcare providers and policymakers in Saudi healthcare system should consider the implementation of early screening for psychological comorbidities and levels of patient adherence to therapies in primary care setting as part of asthma assessment and management plan, considering the high prevalence of anxiety, depression, and poor adherence to controller therapies observed in the current study. This early intervention may eventually prevent further worsening of asthma symptoms which require unnecessary hospitalization as well as treatment escalation (e.g., increasing the dose of ICS and LABA) to keep asthma symptoms under control.

## Conclusion

 Our findings have demonstrated that symptoms of anxiety and depression and poor adherence to asthma medications are prevalent among patients with asthma, and more importantly, we found a relationship between the presence of these psychological symptoms and poor asthma control as well as nonadherence to asthma inhalers. These findings suggest that routine screening for psychological comorbidities and levels of patient adherence to therapies and the implementation of psychoeducational interventions should be considered in clinical practice to identify the presence of psychological symptoms and optimize adherence to therapies early to prevent unnecessary hospitalization and treatment escalation. Further studies are needed to explore the effectiveness of specific psychoeducational interventions and investigate the long-term impact of early psychological symptom detection on asthma outcomes.

### Electronic supplementary material

Below is the link to the electronic supplementary material.

Supplementary Material 1

## Data Availability

All data generated and analyzed during this study are available from the corresponding author upon reasonable request.
