# Supplementary material for: Asthma medication adherence, control, and psychological symptoms: a cross-sectional study
Source: BMC Pulm Med. 2024 Apr 19;24:189. doi: 10.1186/s12890-024-02995-x (PMC11031990; doi:10.1186/s12890-024-02995-x)
Supplement: Supplementary file 1 — Supplementary Material 1 [file 12890_2024_2995_MOESM1_ESM.docx]

Supplementary file

| Variable | Total  (*n* = 287) | |
| --- | --- | --- |
| Depression score (assessed using the HADS tool) | | 2 (IRQ: 1–7) |
| Anxiety score (assessed using the HADS tool) | | 3 (IRQ: 1–8) |
| Adherence to Inhaler score (assessed using the TAI tool) | | 46 (IRQ: 41–49) |
| Asthma control score (assessed using the ACT tool) | | 19 (IRQ: 15–22) |

Median and interquartile range [IQR])
